# Rewiring immune suppression in NSCLC: Roles and plasticity of Tregs and Th17 cells

**DOI:** 10.3389/fimmu.2025.1658848

**Published:** 2025-10-16

**Authors:** Shasha Zhu, Ning Zhou, Qingling Li, Xiaoxing Liu

**Affiliations:** ^1^ Department of Respiratory and Critical Care Medicine, The Affiliated Xuzhou Municipal Hospital of Xuzhou Medical University, Xuzhou, Jiangsu, China; ^2^ Department of Respiratory and Critial Care Medicine, Xuzhou First People's Hospital, Xuzhou, Jiangsu, China

**Keywords:** NSCLC, regulatory T cells, Th17 cells, immunotherapy, tumor immune microenvironment, immune plasticity

## Abstract

Non-small cell lung cancer (NSCLC) exhibits profound immune dysregulation, driven in part by the opposing roles of regulatory T cells (Tregs) and T helper 17 (Th17) cells. Tregs facilitate tumor progression through immune suppression, angiogenesis, and checkpoint engagement, while Th17 cells display dual effects depending on the tumor microenvironment, either promoting anti-tumor responses or enhancing malignancy. Importantly, plasticity between these subsets, orchestrated by cytokines such as TGF-β, IL-6, and IL-1β, allows dynamic interconversion that shapes immune outcomes. This review comprehensively summarizes the differentiation, molecular mechanisms, and functions of Tregs and Th17 cells in NSCLC. We highlight recent advances in targeting the Th17/Treg axis *via* immune checkpoint inhibitors, Treg depletion, and metabolic reprogramming. Understanding this immunological balance offers promising avenues for restoring anti-tumor immunity and improving therapeutic efficacy in NSCLC patients.

## 1 Introduction

Globally, NSCLC continues to be the predominant contributor to cancer-associated deaths, imposing a significant strain on healthcare infrastructures worldwide (1, 2). Recent research has focused on the immunological milieu within tumors, where compromised immune surveillance mechanisms play a pivotal role in oncogenesis and disease advancement (3, 4). Of the diverse immune cell populations, Tregs have been identified as central mediators of immune escape by tumors and are involved in NSCLC (5). Immunosuppression mediated by Tregs occurs *via* cytokine release, interference with metabolic pathways, and direct cytotoxic actions against effector immune cells (6). Conversely, Th17 cells display functional ambivalence, either enhancing or restraining tumor growth contingent upon the inflammatory context (7, 8). This duality is further obscured by the interconversion potential of these subsets, regulated by pivotal cytokines including TGF-β, IL-6, and IL-1β (9).

Beyond their involvement in tumorigenesis, proliferation, dissemination, and metastatic spread, Tregs also collaborate with Th17 cells in the progression of infections, autoimmune conditions, and neoplastic diseases (10). Within NSCLC, accumulating data reveal a complex interaction between Tregs and Th17 cells, both of which significantly influence the immunological profile of tumors (11). Deciphering the equilibrium between Th17 and Treg populations, along with their flexibility, is essential for devising successful immunotherapeutic strategies that reestablish immune homeostasis in NSCLC (12). This review systematically summarizes the differentiation processes, underlying molecular mechanisms, and functional contributions of Th17 and Treg cells in NSCLC. Moreover, it outlines recent breakthroughs in immunomodulatory therapies directed at the Th17/Treg axis, encompassing Treg elimination, inhibition of immune checkpoints, and alterations in cellular metabolism (13), offering novel insights into strategies for overcoming immune suppression and improving clinical outcomes in NSCLC patients.

## 2 Overview of regulatory T cells (Tregs)

### 2.1 Historical identification of Treg lineages

The seminal discovery of regulatory T cells dates to 1995, when Sakaguchi and colleagues demonstrated that selective removal of CD4^+^CD25^+^ T cell populations in murine models triggered systemic autoimmunity, while adoptive transfer of these cells conferred protection, establishing their immunoregulatory function (14). While CD25 serves as an operational surface marker, its expression is not exclusive to this subset (15). The transcription factor FOXP3 has since been identified as both a definitive molecular signature and a master regulator of Treg identity (16). Evidence from genetic analyses underscores FOXP3's non-redundant role in maintaining immunological tolerance, given that loss-of-function mutations precipitate multiorgan inflammatory syndromes across species. The molecular circuitry governing FOXP3 expression involves multiple regulatory layers: The COX-2/PGE2 signaling axis modulates its transcriptional activity, whereas TCR stimulation coupled with CD28-mediated co-signaling induces chromatin reorganization at the Foxp3 gene locus, predominantly through the NF-κB transcription factor c-Rel (17–20). Notably, STAT5-mediated signaling represents an indispensable pathway for the terminal differentiation of FOXP3-expressing Tregs from progenitor populations (21, 22). These molecular mechanisms collectively define the developmental paradigm of Treg specification and continue to inform contemporary models of immune homeostasis.

### 2.2 Functional heterogeneity of Treg populations

Tregs play a pivotal role in sustaining immune tolerance and preventing aberrant inflammatory responses, including those associated with tumorigenesis (23, 24). Unlike antigen-specific immune effectors, Tregs mediate broad immunosuppression, and their functional impairment is linked to autoimmune pathogenesis (25). The transcription factor FOXP3 serves as a critical determinant of their lineage commitment and functional maturation (26). Phenotypically, Tregs are characterized by co-expression of CD25 alongside inhibitory receptors such as CTLA-4, GITR, and LAG-3, as well as membrane-associated TGF-β (27, 28). Further subclassification is possible based on CD45RA expression, distinguishing naïve (CD45RA^+^) from antigen-experienced (CD45RA^-^) subsets (29). Although Neuropilin-1 has been proposed as a potential surface marker, no single definitive identifier currently exists for this population (30). The induced Tregs (iTregs) arise extrathymically from conventional CD4^+^ T cells under specific cytokine milieus, particularly within tumor microenvironments where they paradoxically facilitate immune evasion and malignant progression (31). Among these, Type 1 regulatory T (Tr1) cells—enriched in intestinal mucosa—do not express FOXP3 but instead mediate suppression *via* copious secretion of IL-10 and TGF-β (32, 33). Beyond classical CD4^+^ Tregs, regulatory function extends to multiple lymphocyte lineages. Certain CD4^+^ T cells acquire suppressive properties upon stimulation with autologous dendritic cells, upregulating FOXP3, CTLA-4, and immunomodulatory cytokines (IL-10, TGF-β) (34). Additionally, regulatory activity is observed in innate-like lymphocytes, including IL-10-producing NKT and γδ T cells, as well as CD8^+^CD28^-^ and CD8^+^FOXP3^+^ T cells. Double-negative (CD3^+^CD4^-^CD8^-^) T cells further contribute to immune regulation through analogous mechanisms (35).

### 2.3 Immunosuppressive functions of Treg cells

Tregs mediate immune suppression through diverse mechanisms. Central pathways include the secretion of immunosuppressive cytokines such as TGF-β and IL-10, and the expression of high-affinity IL-2 receptors that deplete IL-2, thereby restricting effector T cell proliferation (36, 37). Soluble factors like IL-10 and TGF-β act in a contact-independent manner. Activated human Tregs also express granzyme A (GZ-A) and utilize the perforin pathway to induce apoptosis in antigen-presenting cells (APCs), while granzyme B contributes to effector T cell suppression (38). The cell-surface repertoire of Tregs features several co-inhibitory molecules essential for their function. Notably, CTLA-4 and GITR engage cognate receptors on target cells to transmit inhibitory signals, with CTLA-4 additionally facilitating the induction of regulatory phenotypes in CD4^+^ T cell precursors (39, 40). Besides, other critical regulators include PD-1, LAG-3, and CD39. LAG-3 modulates APC activity through MHC class II interaction, PD-1/PD-L1/PD-L2 signaling promotes Foxp3^+^ Treg development (41), while CD39 generates immunoregulatory adenosine *via* nucleotide catabolism (42). The suppressive arsenal of Tregs extends to metabolic interference through IDO-dependent tryptophan degradation, cytotoxic effector mechanisms involving perforin/granzyme systems (43, 44), and suppression of NK cell-mediated cytotoxicity by interfering with NKG2D signaling pathways (45). Their multifaceted regulation operates across immunological contexts through spatial competition with naïve T cells for APC engagement *via* chemokine gradients, and functional impairment of dendritic cell maturation; dynamic secretion of IL-10, IL-35, and cytotoxic mediators tailored to microenvironmental cues.

## 3 Properties of Th17 and Treg cells

Th17 cells constitute a unique CD4^+^ T helper subset, distinct from classical Th1 and Th2 lineages. Harrington identified IL-17–producing CD4^+^ T cells in mice, which were subsequently termed Th17 cells (46). Lineage-defining transcriptional regulators RORγt and STAT3 govern both their developmental program and functional stability (47, 48). Th17 lineage commitment is highly dependent on the cytokine environment. IL-6 and TGF-β act cooperatively to promote Th17 polarization (49). The concentration of TGF-β is crucial in determining CD4^+^ T cell fate, lower levels favor RORγt expression and Th17 differentiation, while higher levels suppress RORγt and induce Foxp3, promoting Treg development (50, 51). Notably, IL-21 can substitute for IL-6 in the presence of TGF-β to induce RORγt and inhibit Foxp3, further facilitating Th17 differentiation (51). Functionally, Th17 cells are pro-inflammatory, primarily through the secretion of IL-17, their hallmark cytokine (52, 53). The Th17/IL-17 axis has been implicated in autoimmune diseases such as asthma, systemic lupus erythematosus, and rheumatoid arthritis, although its role in tumor biology remains controversial and under active investigation (53–55).

Tregs are essential mediators of immune tolerance and immune suppression in both physiological and pathological contexts, including tumor immunity (56). Although Tregs constitute a minor fraction of CD4^+^ T lymphocytes, their capacity to suppress effector T cell responses enables tumors to evade immune surveillance in NSCLC (57, 58). The transcription factor FOXP3 remains a key determinant of Treg identity and function, exerting transcriptional repression of pro-inflammatory genes to encode inflammatory mediators such as IFN-γ, IL-13, and GM-CSF (59–61). In addition, Tregs modulate dendritic cell activity by secreting immunosuppressive cytokines such as IL-10, which promotes DC apoptosis and impairs their antigen-presenting capacity by downregulating co-stimulatory molecules like CD80 and CD86 (62, 63). These effects reduce effective T cell priming and promote an immunosuppressive microenvironment (64). Importantly, in NSCLC, Tregs express high levels of PD-1, CTLA-4, and CD39, which contribute to immune checkpoint-mediated suppression and adenosine production that further dampens effector cell functions (65–67). By shaping the tumor immune landscape through direct suppression and immune modulation, Tregs play a pivotal role in promoting tumor progression and resistance to immunotherapy.

## 4 Roles of Th17 and Treg cells in NSCLC

The immunosuppressive TME in NSCLC is particularly pronounced, characterized by high infiltration of Tregs, chronic inflammation, and resistance to immune checkpoint blockade therapies (68). Notably, NSCLC has been extensively studied in Th17 and Treg cell dynamics, offering a well-established framework to investigate their functional plasticity and therapeutic implications (69). Given these features, NSCLC represents a clinically relevant and immunologically tractable model for dissecting the Th17/Treg axis.

### 4.1 Roles of Th17 cells in NSCLC

**Figure 1:**
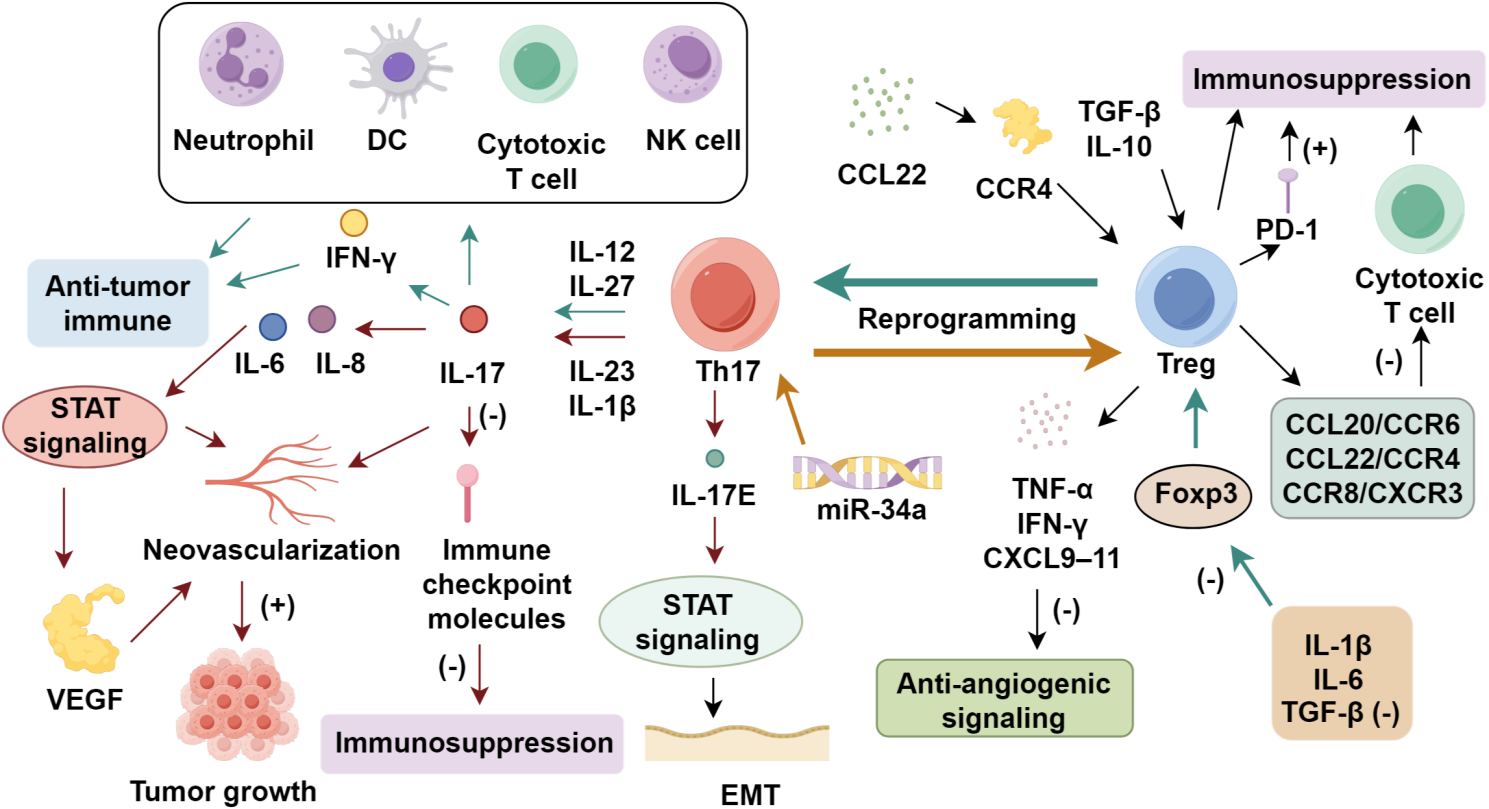
The role of regulatory T cells in non-small cell lung cancer progression.

The functional dichotomy of Th17 cells in NSCLC continues to be a subject of controversy, as these lymphocytes exhibit both pro-tumorigenic and anti-tumor activities (70). Elevated concentrations of IL-17, a key Th17-derived cytokine, correlate with augmented neovascularization in multiple malignancies, suggesting a role in facilitating tumor growth (71). Huang et al. (72) reported that IL-17 increased microvessel density and VEGF *via* STAT signaling, upregulating IL-6 and IL-8. IL-17 administration accelerated tumor growth in mice (73). In contrast, increased Th17 infiltration within the tumor microenvironment has been shown to coincide with elevated neutrophil recruitment alongside heightened IFN-γ secretion, implying a capacity to bolster anti-tumor immune responses (74, 75). Ye et al. (76) revealed a marked enrichment of Th17 cells in NSCLC-associated malignant pleural effusions compared to peripheral blood, with higher Th17 frequencies predicting improved patient outcomes. The lineage-defining transcription factor RORγt orchestrates Th17 differentiation and may facilitate their transdifferentiation into cytotoxic CD8^+^ T lymphocytes. Additionally, RORγt-driven IL-17 production has been implicated in the suppression of immune checkpoint molecules, potentially mitigating tumor-induced immunosuppression (77). In NSCLC, IL-17E facilitates cell proliferation and epithelial-mesenchymal transition in A549 cells by regulating the NF-κB pathway (78). Th17 cells activate dendritic cells, enhance effector and cytotoxic T cell responses, and promote NK cell infiltration, collectively strengthening anti-tumor immunity (79, 80). The dual behavior of Th17 cells is largely shaped by the upstream cytokine milieu and the tumor microenvironmental context. IL-23 and IL-1β are critical determinants of Th17 pathogenicity (81, 82). IL-23 stabilizes the Th17 phenotype and promotes expression of pro-tumor mediators such as IL-17A, IL-22, and GM-CSF, while inhibiting anti-tumor features such as IFN-γ production (83–87). IL-1β, in cooperation with IL-6 and low-dose TGF-β, biases Th17 cells toward a pathogenic profile that favors inflammation, angiogenesis, and tumor progression (13, 88). In contrast, IL-12 or IL-27 exposure can redirect Th17 cells toward an IFN-γ–producing, tumoricidal phenotype (89) (**Figure 1**).

### 4.2 Roles of Tregs in NSCLC

#### 4.2.1 Tregs in NSCLC initiation and progression

In early stage, the immune system maintains equilibrium by eliminating spontaneously arising tumor cells through coordinated innate and adaptive immune responses (90). However, when malignant cells proliferate beyond the capacity of immunological control mechanisms, this homeostatic balance is disrupted, leading to impaired immune surveillance and functional deficits (91). Such disruption enables immune escape and promotes malignant phenotypes, including unchecked proliferation, genomic instability, and metastasis. Among these, CD4^+^CD25^+^ Tregs have been increasingly recognized as critical mediators of immune suppression in NSCLC (14). Clinically, increased Treg frequencies are consistently observed in both tumor sites and peripheral blood of lung cancer patients (92). This expansion is orchestrated by the tumor microenvironment, where immunosuppressive cytokines such as TGF-β and IL-10 induce naïve T cell conversion into Tregs, and chemokines like CCL22 mediate recruitment *via* CCR4 signaling (93). Infiltrating Tregs then reinforce immunosuppression, forming a feedback loop that accelerates immune escape (94). Moreover, TGF-β–induced Treg infiltration suppresses cytotoxic T cell activity in NSCLC. These include chemokine-mediated recruitment *via* CCL20/CCR6, CCL22/CCR4, CCR8, and CXCR3 (95, 96), antigen-driven clonal expansion facilitated by dendritic cell presentation and TGF-β–dependent polarization (96), metabolic reprogramming favoring glycolytic and lipid oxidation pathways to support Treg survival (97, 98), and the contribution of tumor-derived extracellular vesicles that enhance Treg proliferation and confer resistance to apoptosis (99, 100).

#### 4.2.2 Tregs in invasion and metastasis of NSCLC

Elevated Treg levels are strongly associated with advanced clinical stage, poor differentiation, and enhanced metastatic potential in lung cancer (68). Prognostic analyses consistently identify tumor-infiltrating Treg abundance as an independent predictor of unfavorable clinical outcomes. These immunosuppressive cells promote metastatic progression through diverse biological pathways (101, 102). Tregs disrupt anti-angiogenic signaling by inhibiting Th1-cell derived mediators including TNF-α, IFN-γ, and CXCL9–11 (103, 104). Hypoxia further induce the VEGF production, fostering tumor vascularization (105, 106). The stromal compartment contributes to therapy resistance through elevated COX-2/PGE2 pathway activity, which simultaneously enhances Treg differentiation and metastatic potential (107, 108). Functionally, Treg-mediated immune suppression manifests through impaired CD8^+^ T cell cytotoxic activity, with experimental depletion studies demonstrating restored expression of effector molecules (perforin, granzyme) and Th1 cytokines (109, 110). Clinically, elevated TGF-β and IL-10 in circulation and tumor tissues reflect Treg-mediated immunosuppression (111). Foxp3^+^ Tregs are increased in patient blood and decline postoperatively, implicating them in tumor development (112). Notably, Tregs engage in functional crosstalk with immune checkpoint pathways, particularly through their high PD-1 expression, which appears to amplify immunosuppressive activity and promote immune evasion (113). This mechanistic insight has spurred the development of several Treg-targeted therapeutic strategies. These include novel anti-CD25 antibodies like RG6292, which is engineered to deplete immunosuppressive Tregs while sparing IL-2 signaling in effector T cells, as well as combination approaches that integrate immune checkpoint inhibitors with Treg-targeting agents, currently under evaluation (114). These next-generation approaches demonstrate improved specificity and reduced toxicity profiles compared to earlier agents such as diftitox (115). Additionally, metabolic reprogramming remains a promising adjunctive strategy, with S-adenosylmethionine (SAM) showing potential to modulate Treg plasticity by downregulating Foxp3 and IL-10 while simultaneously enhancing IFN-γ production (116).

### 4.3 Dynamic interplay between Th17 and Treg cells in NSCLC pathogenesis

The functional plasticity between Th17 and Treg populations represents a critical immunoregulatory mechanism in NSCLC, with these cell subsets demonstrating capacity for bidirectional conversion that dynamically shapes tumor immunity (13, 69). Cytokines such as IL-1β and IL-6, secreted predominantly by tumor-associated macrophages and stromal cells, in concert with suboptimal concentrations of TME-derived TGF-β, drives Treg-to-Th17 reprogramming through Foxp3 suppression and impairment of regulatory function (117, 118). This phenotypic switching involves Treg acquisition of c-like properties, characterized by ROR-γt upregulation, Foxp3 loss, and development of IL-17 secretory capacity (119, 120). Post-transcriptional regulation further modulates this plasticity, as demonstrated by miR-34a-mediated enhancement of Th17 differentiation coupled with Treg functional inhibition (121). Clinically, NSCLC patients exhibit concurrent elevation of both subsets in circulation, with the Th17/Treg ratio serving as a more informative immunological parameter than absolute cell counts (13, 122). For example, Li et al. demonstrated that NSCLC patients displayed a significant increase in the Th17/Treg ratio post-treatment, suggesting its potential utility as a predictive marker of therapeutic efficacy (13). This ratio demonstrates stage-dependent progression, showing positive correlation with advancing tumor burden (123). Notably, this balance shifts throughout tumor progression: early-stage NSCLC, characterized by low TGF-β and high IL-6, favors Th17 polarization, whereas advanced stages, enriched in TGF-β, promote Foxp3 expression and Treg dominance (124–126). Crucially, the immunological impact of Th17 cells is not defined by their absolute numbers alone but by their dynamic balance with Treg cells. This Th17/Treg interplay determines the net immune response toward either tumor suppression or promotion in NSCLC (**Supplementary Table S1**).

## 5 Conclusion

The intricate interplay between Tregs and Th17 cells represents a central axis of immune regulation in non-small cell lung cancer (NSCLC). Tregs suppress anti-tumor immunity through cytokine secretion, checkpoint engagement, metabolic modulation, and inhibition of cytotoxic effector cells, thereby promoting tumor immune evasion, angiogenesis, and metastasis. In contrast, Th17 cells display context-dependent functions—exerting either tumor-promoting or tumor-inhibiting effects depending on the cytokine milieu, tumor stage, and metabolic cues within the tumor microenvironment. The dynamic balance and plasticity between these two subsets, particularly their bidirectional interconversion mediated by TGF-β, IL-6, and IL-1β, critically shape the immune landscape of NSCLC.

Targeting the Th17/Treg axis offers a promising strategy to restore immune surveillance and improve therapeutic responses in NSCLC. Advances in Treg-selective depletion, immune checkpoint inhibition, and modulation of T cell differentiation through metabolic or epigenetic interventions provide novel avenues for immunotherapy. Future research should prioritize refining these approaches, optimizing combination regimens, and identifying predictive biomarkers such as the Th17/Treg ratio to guide individualized treatment. A better understanding of the functional plasticity between Tregs and Th17 cells will be essential to overcoming immunosuppression and enhancing durable responses in NSCLC patients.
